# Stigmatising attitude of medical students towards a psychiatry label

**DOI:** 10.1186/1744-859X-7-15

**Published:** 2008-08-25

**Authors:** Olawale O Ogunsemi, Olatunde Odusan, Michael O Olatawura

**Affiliations:** 1 Olabisi Onabanjo University Teaching Hospital, Sagamu, Ogun State, Nigeria

## Abstract

**Background:**

The aim of this study is to evaluate the effect of a psychiatric label attached to an apparently normal person on the attitude of final year medical students at a Nigerian university.

**Methods:**

A questionnaire with sections on demographic information, a single-paragraph case description illustrating a normal person, a social distance scale and questions on expected burden was used to elicit responses from 144 final year medical students who have had previous exposure to psychiatric posting. The students consisted of two randomly assigned groups; group A received a case description with a psychiatric label attached while group B received the same case description but without a psychiatric label.

**Results:**

A total of 68 (47.2%) of the students responded to the questionnaire with the attached psychiatric label, while 76 (52.8%) responded to the questionnaire without the attached label. There was no statistical difference in age (p = 0.187) and sex (p = 0.933) between the two groups of students. The students who responded to the questionnaire with the attached psychiatric label would not rent out their houses (p = 0.003), were unwilling to have as their next-door neighbour (p = 0.004), or allow their sister to get married (p = 0.000) to the man depicted in the case description compared with those that responded to the questionnaire without label. This group also felt that the man would exhaust them both physically (p = 0.005) and emotionally (p = 0.021) in any relationship with him.

**Conclusion:**

These results strengthen the view that stigma attached to mental illness is not limited to the general public; medical students are also part of the stigmatising world. There is, therefore, a need to incorporate issues concerning stigma and its reduction as a core component of the mental health curriculum of medical schools.

## Introduction

In most societies mental illness carries a substantial stigma [1,2]. The mentally ill are often blamed for bringing on their own illnesses, while others may see them as victims of unfortunate fate, religious and moral transgression, or even witchcraft. Such stigma may lead to denial on the part of the family that one of their members is psychiatrically ill. Some families may hide or overprotect a member with mental illness, thus keeping the person from receiving potentially effective care.

Stigma remains a powerful negative attribute in all social relations. It is considered as an amalgamation of three related problems: a lack of knowledge (ignorance), negative attitudes (prejudice), and exclusion or avoidance behaviours (discrimination) [3,4].

The mentally ill are labelled as different from other people and are viewed negatively by others. Stigmatisation can lower a person's self esteem, contribute to disrupted family relationships, and affect employability [5]. It is a barrier to the provision of mental health services by health planners [6].

Many studies have demonstrated that persons labelled as mentally ill are perceived with more negative attributes and rejection regardless of their behaviour [7-9]. Research has shown that people who are labelled as mentally ill associate themselves with society's negative conceptions of mental illness, and that society's negative reactions contribute to the incidence of mental disorder. [10]. However, other studies have demonstrated that negative societal reactions are the result, rather than the cause, of mental illness [11].

Individuals who perpetuate stigma are likely to socially distance themselves from persons with mental illness. Social distance may manifest itself in such discriminatory practices as, for example, not renting property to or hiring people who have psychiatric disabilities [5,12].

Stigmatising views about mental illness are not limited to uninformed members of the general public; even well-trained professionals from most mental health disciplines subscribe to stereotypes about mental illness [13,14]. Medical students have been shown to have stigmatising attitudes toward mental illness which they hold onto in their professional lives [15]. Therefore, research on attitudes toward mental illness, specifically of those in mental health related fields, is necessary to ensure quality care to persons with mental illness. This is important because interventions directed at these target groups may be more cost effective than interventions directed at the general public [16].

This study aimed to evaluate the effect of a psychiatric label attached to an apparently normal person on the attitude of final year medical students in a Nigerian university.

## Methods

This was a cross sectional questionnaire based study conducted among the final year medical students of Olabisi Onabanjo University, Ogun State. Participation was on a voluntary basis. A questionnaire containing demographic information, a single-paragraph case description illustrating a normal person, a social distance scale and questions on expected burden was used to elicit response from the students.

The students were randomly assigned into two groups using their matriculation numbers. Group A received a case description with a psychiatric label attached while group B received the same case description but without a psychiatric label.

The case description is as follows 'Mr AB is a young man who can express his feelings and thoughts among those close to him, although he sometimes gets anxious while talking in a group consisting of strangers. He gets along all right with his family most of the time. Generally he also gets along with other people. Compared to those of his age, his life can be considered as organised. He is generally an optimistic and happy person. In summary, he establishes a good balance between his social life and study'. The students were assigned to one of the two conditions of the case description. One condition involved adding the sentence 'This young man has been diagnosed as having mental illness by the doctor who examined him' to the end of the case description. In the second condition no psychiatric label was attached to the case description.

Each case description was followed by 16 questions to be rated on a 4-point scale ranging from definitely agree to definitely disagree. The questions from 1 to 13 were designed to measure social distance between oneself and the person depicted in the case description while questions 14, 15 and 16 assess the possible burden expected from a mentally ill person one may associate with. The case description and the questionnaire were modified versions of those used in previous studies that concerned psychiatric label and attitude to mental illness [17].

The data derived from the responses of the students were analysed using SPSS v.10 (SPSS Inc., Chicago, IL, USA). Results are presented in frequencies and percentages. The Chi square test was used to determine statistical difference between proportions while the Student t test was used to determine the statistical difference between means. A p value less than 0.05 was considered as statistically significant.

## Results

A total of 144 students responded to the questionnaire out of a class of 167. Thus, the response rate was 86.2%. In all, 81 (56.2%) were males while 63 (43.8%) were females. A total of 68 (47.2%) of the students responded to the questionnaire with the attached psychiatric label (male 55.9%, female 44.1%) while 76 (52.8%) responded to the questionnaire without the attached psychiatric label (male 56.6%, female 43.4%) (p = 0.933). The mean (SD) age of the students that responded to the questionnaire with the attached psychiatric label was 27.07 (3.33) years compared with 26.96 (2.18) years for those that responded to the questionnaire without the psychiatric label (p = 0.187).

Table 1 shows the responses of the students to the questions about the man depicted in the case description. The students that responded to the questionnaire with the attached psychiatric label were significantly more unwilling to rent out their houses to the man depicted in the case description compared to those that responded to the questionnaire without the attached label (p = 0.003). Similarly, they were unwilling to have him as their next-door neighbour (p = 0.004) or have him as their barber or hairdresser (p = 0.000) compared with the group that responded to the questionnaire without the attached label. They were also not willing to share an office with him (p = 0.000) or allow their sister to get married to him (p = 0.000).

**Table 1:** responses of the students to the person depicted in the case description

	**Label attached** ** frequency, % (n = 68)**	**No label attached:** ** frequency, % (n = 76)**	**p Value**
Uncomfortable sitting close to him on public transport	36 52.9	31 40.8	0.144
Disturbed by shopping from a market which he runs	13 19.1	16 21.1	0.774
Willing to let your house to him	39 57.4	61 80.3	0.003
Ill at ease by his working as a gateman at your house	31 45.6	25 32.9	0.112
Disturbed participating in a social gathering to which he has been invited	13 19.1	20 26.3	0.308
Willing to play cards with him at a social gathering	54 79.4	50 65.8	0.087
Willing to chat with him on political matters at a social gathering	41 60.3	49 64.5	0.614
Willing to tell him about your own private problems	19 27.9	27 35.5	0.305
Disturbed by his becoming your next-door neighbour	20 29.4	08 10.5	0.004
Will have my hair cut/styled by him if he was a barber/hairdresser	26 38.2	57 75.0	0.000
Disturbed by working in the same place as him	03 04.1	05 06.6	0.616
Will be worried sharing the same room with him if you work at the same place	34 50.0	12 15.8	0.000
Disturbed by your sister wanting to marry him	49 72.1	28 36.8	0.000
Will be an emotional burden on you in your friendship with him	17 25.0	09 11.8	0.021
Will exhaust your physical energy in your friendship with him	20 29.4	09 11.8	0.005
Your friendship with him will have a negative influence on your mental health	08 11.7	10 13.2	0.884

Significantly, the students that responded to the questionnaire with the attached label felt that the man in the case description will exhaust them both physically (p = 0.005) and emotionally (p = 0.021) in their relationship with him, compared with those that responded to the questionnaire without the psychiatric label.

## Discussion

This study set out to investigate the effect of a psychiatric label attached to an apparently normal person in a case description on the attitude of final year medical students toward psychiatrically ill patients. The students have had previous clinical exposure to psychiatry in the course of their medical training. The finding in this study indicated that a label of mental illness on the person depicted in the case description elicited negative attitude that resulted in the students wanting to maintain a significant distance from the person that was labelled mentally ill. The results provided strong support for the influence of labelling on certain attitudes. These attitudes were more obvious in circumstances that could bring a closer relationship between the respondents and the person depicted in the case description. They were not willing to have him as their barber/hairdresser, they would discourage their sister from planning to get married to him, and they were uncomfortable with the thought of sharing an office with him. These findings are consistent with previous studies on the influence of psychiatric label on attitude towards mental illness [8,9,17]. Furthermore, the students felt that friendship with the labelled person would be a burden on them physically and emotionally. This could further worsen the social distance between them and the labelled person. These stigmatising attitudes have been shown to increase psychological distress in people labelled to be mentally ill [18]. Moreover such attitudes may inhibit help seeking among individuals with a mental disorder [19,20] and provide barriers to their successful reintegration into the society [21].

The findings in this study provide support for an earlier report by Adewuya and Makanjuola [22] on the attitudes of students generally toward the mentally ill in a Nigerian university. This however, challenges studies where less stigmatisation of mental illness was reported for non-Western cultures especially of Asian and African countries [23,24]. Although a dearth of research on this issue was given for the observation in these cultures, Fabrega, however, noted that lack of differentiation between psychiatric and non-psychiatric disorders in non-Western cultures could be an important factor for less stigmatisation [23,24]. It is however important to note that this study was conducted in a group of students who are medically inclined, and hence they should be able to differentiate between psychiatric and non-psychiatric disorders.

Although a considerable number of studies have consistently reported improvement in attitude of medical students toward psychiatry after clinical exposure [25,26], follow-up studies on such students have queried the sustenance of the observed improvement in attitude toward psychiatry over time even before they eventually graduate from medical school [27]. Thus, the finding of a stigmatising attitude of the final year medical students in this study may be a reflection of this decay. The challenge then will be to find a way of sustaining the initial improvement reported in the literature. The focus will be to provide a more cost effective approach of educating the medical students on stigma reduction in mental health. This is particularly important because stigma involves different but related facets [3,4]. This is however, hindered by deficiencies in the mental health curriculum in medical schools where little or no attention is given to stigma as an issue. Moreover, the common medical textbooks in psychiatry fail to devote elaborate attention to issues on stigmatisation of mental illness.

In conclusion, medical students are not exonerated from the list of people that express stigmatising attitude toward the mentally ill. There is therefore the need to equip the students with more knowledge on stigma reduction in mental health.

## Competing interests

The authors declare that they have no competing interests.

## Authors' contributions

OOO conceived the study, and participated in its design, acquisition, analysis and interpretation of data, and in the drafting of the manuscript. OO participated in its coordination, statistical analysis and helped to draft the manuscript. MOO participated in the design of the study, its coordination and the draft of the manuscript. All authors read and approved the final manuscript.
